# Enhancing Conservation of a Globally Imperiled Rockland Herb (*Linum arenicola*) through Assessments of Seed Functional Traits and Multi-Dimensional Germination Niche Breadths

**DOI:** 10.3390/plants9111493

**Published:** 2020-11-05

**Authors:** Héctor Eduardo Pérez, Luis Andres Ochoa Chumana

**Affiliations:** 1 Department of Environmental Horticulture, University of Florida, 2047 IFAS Research Drive, Gainesville, FL 32611, USA; lochoac@unemi.edu.ec; 2 Ministerio de Agricultura y Ganadería, Unidad de Gestión Distrital de Desarrollo Productivo, 24D01, Calle 10 de Agosto y Juan Montalvo, Santa Elena-Santa Elena, Ecuador

**Keywords:** endangered, environmental gradients, niche breadth, non-deep physiological dormancy, oolitic limestone, photoperiod, sand flax, temperature, salinity

## Abstract

Humans currently face an extraordinary period of plant biodiversity loss. One strategy to stem further losses involves the development of species-level recovery plans that guide conservation actions. Seeds represent an important component in the life history of plants and are crucial for conservation activities. Yet, most recovery plans contain meager seed biology information. We set out to examine seed functional traits and germination niche breadth of *Linum arenicola* seeds exposed to a range of thermal, photoperiodic, and salinity gradients to gain perspectives on the seed biology of this endangered species that may inform conservation decision making and assist recovery plan development. We found that fresh seeds possess non-deep physiological dormancy, which may be alleviated via a four-week dry after-ripening treatment. The germination response of non-dormant seeds is subsequently promoted by constant rather than alternating temperatures. The optimum germination temperature range is 20–22 °C. Non-dormant seeds do not possess an absolute light requirement for germination, but are sensitive to low levels of salinity (EC_50_ = 6.34 ppth NaCl). The narrow thermal and salinity germination niche breadths reported here suggest a specialized reproductive strategy that may require careful consideration when planning ex and in situ conservation activities.

## 1. Introduction

Species extinctions measured on geologic time scales are a normal part of the evolutionary process. Pimm et al. [1] estimate that the background extinction rate due to naturally occurring environmental and ecological factors is about 0.1 extinctions per million species years (E/MSY). This stands in stark contrast to the estimated 1000-fold increase in current extinction rates induced by human activity. Current extinction rates may vary (mean ~100 E/MSY) by region and species [1]. However, plants seem disproportionately at risk. Current estimates suggest that almost 33 to 40% of all plant species are threatened with extinction [2,3]. Such information is startling considering that humans depend on plants for life and points to an unprecedented time of plant biodiversity loss.

Various governmental and non-governmental organizations recognize the global extinction crisis and endeavor to curtail plant biodiversity loss through various conservation strategies. One method focuses on species-level conservation activities often guided by recovery action plans [4,5,6,7]. The intent of these plans is to provide guidance for management actions needed to recover threatened species. Therefore, sufficient species level biological information is necessary to formulate and implement prescribed recovery goals. Additionally, the amount and recency of species biological information included within action plans influences recovery outcomes [8,9].

Plant seeds provide many advantages compared with whole plants, or plant parts, that may facilitate conservation activities. For instance, maternal plants may produce an abundance of seeds that are relatively easy to collect, handle, and transplant. The comparatively small size of seeds increases the density of stored propagules per storage area. Likewise, small seed size can facilitate the establishment of soil seed banks. Furthermore, seed desiccation tolerance is extensive among the spermatophytes [10]. Expression of this trait enables long-term seed storage within ex situ conservation collections. These are some reasons practitioners place an emphasis on the use of seeds for in and ex situ recovery actions [11,12,13,14].

The increased attention on seed functional trait [15,16] and germination niche [17] based ecological research also provides key biological information that may enhance recovery plans. For example, examination of seed traits like response to chemical cues, germination speed, light and temperature requirements for germination, and stress tolerance can help characterize important seed functions such as persistence, germination timing, and establishment, which in turn assist in delineating ecological strategies used by species to assemble populations and communities [15,16].

Grubb [17] described the regeneration niche as a manifestation of conditions necessary for one mature individual of a subsequent generation to replace a mature individual of a previous generation with a high likelihood of success. Sexton et al. [18] extend this by defining niche breadth as the mix of resources, habitats, or environments used by a given species. Germination is one component of the regeneration niche and measuring germination performance across environmental gradients provides a basis for calculating germination niche breadth. The available ranges of temperature, light conditions, chemical compounds, and moisture cues represent important environmental niche dimensions [18] that define germination niche breadth. Calculated germination niche breadths thus provide information on the degree of specialization (i.e., narrow niche) or generalization (i.e., broad niche) of abiotic and biotic resources used by a species to enable transition from the seed to seedling life history stage.

Specialization tends to be favored by species occurring in temporally stable environments (e.g., warm, tropical climates), while temporally unstable environments (e.g., temperate climates) tend to promote a generalist strategy [18,19,20,21]. Additionally, niche breadths are known to vary among populations and within succeeding generations of a population [22,23]. Clarification of a species germination niche breadth has implications for other questions related to determination of species diversity in a given area, similarities between species and relationships to coexistence, partitioning of an environment between species, how species within a community shape inter-species evolution, and adaptation of individuals at the population level [18,20].

Species must contend with various environmental gradients to regenerate. Seeds, especially those in the hydrated state, act as superb environmental sensors consistently integrating abiotic signals to maximize the probability of germination, leading to successful seedling [24,25,26,27,28,29]. Temperature is a key abiotic signal that regulates germination and may serve as cues for sensing seasonality and vegetation gaps. Non-dormant seeds are capable of germinating over a wide temperature range. Seeds of some species display preferential germination under constant rather than alternating temperatures regimes. However, seed germination response is typically higher under fluctuating rather than constant temperatures. Likewise, some species may have seeds with an absolute requirement for exposure to light to complete germination, while other species possess seeds that are sensitive to light and require darkness for germination. Yet, seeds of other species are indifferent and can germinate under either condition [24,30].

Soil salinity may also have a profound effect on germination and seedling establishment. Low levels of salinity typically inhibit germination for species not adapted to saline environments. However, halophytic and psammophytic species from various habitats display inconsistent germination sensitivity across a breadth of salt concentrations. For example, germination of salt marsh and salt desert species is reduced to about 10% when salt concentrations range from around 2.8 to 100 parts per thousand (ppth, mean = 22 ppth). Reduction of germination to low levels (≤10%) for species occurring on beaches, cliffs, and foredunes corresponds to salt concentrations between 3.5 to 35 ppth with a mean of 19 ppth [24,30].

Examining multiple niche dimensions elucidates a set of environmental conditions in which a species can survive and reproduce [18]. Therefore, seed functional trait and multi-dimensional germination niche breadth data can inform conservation practice and enhance species recovery plans at scales from ex situ propagation to in situ seedling establishment in a target habitat. Unfortunately, many recovery plans contain minimal to no seed biology information for listed species and conservation programs in general require an expanded seed biology knowledge base [31].

Plant conservation programs, and associated species recovery plans, need an infusion of seed biology information. This problem is especially acute for species recently listed by agencies to receive protected status [8,9]. *Linum arenicola* (Small) H.J.P Winkler (sand flax, Linaceae) is a globally imperiled species that achieved federally protected status in 2016 [32]. Some preliminary work suggests that seeds of *L. arenicola* may be amenable to long-term storage in genebanks [33,34] and persist in sandy soil for up to nine months [33], but otherwise, no further seed biology information has been reported. Additionally, a recovery plan for *L. arenicola* is not yet available. Consequently, an opportunity exists to provide seed biology data specific to *L. arenicola* with the potential to inform conservation planning.

*Linum arenicola* is a small, perennial herb endemic to oolitic limestone rocklands of the extreme southeastern tip of the Florida peninsula (Miami-Dade County) and the Florida Keys (Monroe County). Fourteen occurrences of *L. arenicola* have been mapped between Miami-Dade and Monroe counties. It is estimated that roughly 10,000 individual plants exist in remnant habitat patches, but 10–30% of these individuals occur in non-natural settings. The long-term outlook for this species estimates a 50–70% decline of individuals [35].

The rocklands on which *L. arenicola* occur are also globally imperiled [36]. This fire-maintained ecosystem is characterized by exposed, well-drained limestone that often forms outcrops. The soil profile ranges from shallow (e.g., 8–15 cm) sandy or marl type soils over a limestone layer in some locations to mostly non-existent with loose rocky rubble on the surface. Organic matter can accumulate in crevices and solution holes formed via dissolution of upper limestone layers. The actual rooting medium that forms in solution holes can contain about 30–50% organic matter, while surface soils, if these exist, typically contain ≤10% organic matter [37]. *Linum arenicola* is found mostly in open, exposed areas within rocklands, but may also occur in solution holes [36].

Factors including habitat fragmentation, altered hydrology and fire regimes, invasive plant species, and habitat conversion threaten *L. arenicola* and rocklands. More specifically, altered disturbance regimes and invasive plant species encroachment can produce shifts in microclimatic temperatures and light availability, which in turn directly influence germination dynamics [24,38]. Similarly, sea level rise and changes in soil salinity pose major threats throughout the *L. arenicola* range. For example, the water table is typically within 25 cm of the soil surface throughout southeastern Miami-Dade county and salt water intrusion has been identified at least 6 km inland of Biscayne Bay [39]. The area of saltwater intrusion overlaps several occurrences of *L. arenicola*. Likewise, *L. arenicola* occurs along the banks of a levee-canal flood control system (i.e., the L-31E canal, Figure S1) immediately adjacent to Biscayne Bay. Plants growing along the L-31E levee banks represent the largest *L. arenicola* population. Rising sea levels, extreme high tide events, and malfunctioning water flow control valves have converted the L-31E canal from a fresh water to saline water body. Surface water salinity levels in the L-31E canal range from 4.3 to 30 ppth. On several occasions, tidal inflow and extreme high tide events have led to significantly elevated water levels in the L-31E canal and along the levee banks [40]. Ross et al. [41] also report soil salinity measurements ranging from 5 to 14 ppth in the lower Florida Keys. Bradley and Saha [42] conclude that adult *L. arenicola* plants are susceptible to inundation and the resulting high soil salinity levels brought on by tropical cyclone produced storm surges. However, the influence of salinity on seed germination is not known.

Our aim here is twofold. First, we set out to examine the germination ecology of *Linum arenicola* using key seed functional traits and assess the degree of germination niche breadth in response to thermal, saline, and light cues. Next, we translate this information to enhance conservation strategies for this species.

## 2. Results

### 2.1. Germination Capacity under Constant or Simulated Seasonal Temperatures

No germination occurred at any temperature for fresh seeds removed immediately from fruits collected in 2017. Likewise, no germination occurred for seeds exposed to 6, 31, 36, or 40 °C from capsules collected in 2017 and stored four weeks prior to the experiment. Germination at the remaining temperatures for seeds harvested following fruit storage ranged from about 60 to 100%. Germination rate peaked and was 1.4 to 3.5 times faster for seeds exposed to 22 °C compared with the remaining temperatures (Table 1).

**Figure 1:**
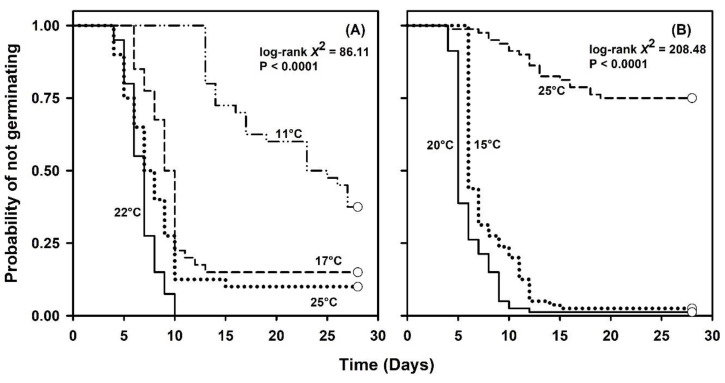
Kaplan–Meier estimates of survivor functions for *Linum arenicola* seeds exposed to constant temperatures of (**A**) 11, 17, 22, or 25 °C or (**B**) 15, 20, or 25 °C. Pointwise 95% confidence intervals omitted for clarity.

Temporal patterns indicated a germination lag of 5 to 6 d for seeds exposed to 17 to 25 °C. However, germination lag was about twice as long for seeds exposed to 11 °C. All germination events occurred by day 15 for seeds at 17 to 25 °C (Figure 1A). However, germination was over two times less uniform as temperature increased from 17 or 22 to 25 °C (Table 1). Alternatively, the temporal pattern of germination for seeds exposed to 11 °C was much less uniform than at higher temperatures (Figure 1A). Moreover, the probability of not germinating decreased continually for seeds exposed to 17 to 25 °C. The null hypothesis that survivor functions were the same across constant temperatures was rejected (log-rank $\chi_{3}^{2}$ = 86.11, *p* < 0.0001).

Cox regression detected a highly significant constant temperature effect on post-storage germination (Wald *χ*^2^ = 36.69, *p* < 0.0001). The likelihood of germination increased by 51% for every 1 °C increase in temperature above 11 °C (Table 2). Linear contrasts revealed strong differences in the likelihood of germination between 11 °C and the remaining temperatures. However, the largest differences in the likelihood of germination within a group of temperatures with the same minimum temperature appeared for contrasts against 22 °C (Table 3).

No seeds from the 2018 lot germinated at 10, 30, or 35 °C. However, the final germination percentage for seeds exposed to 15 or 20 °C was greater than 97%. Final germination declined by about 74% for seeds treated at 25 °C. The germination rate was nearly 1.3 times more rapid at 20 °C compared with 15 °C (Table 1). Temporal patterns of germination revealed a lag of about 4 to 6 days for seeds at 15 or 20 °C. One seed exposed to 25 °C germinated at day four, but germination for most seeds commenced around day seven. Seeds completed germination between day 12 and 15 following exposure to 15 or 20 °C and uniformity appeared similar between these treatments. Alternatively, seeds at 25 °C displayed much less germination uniformity than at remaining temperatures (Figure 1B). The null hypothesis of homogeneous survivor functions was rejected (log-rank $\chi_{2}^{2}$ = 208.48, *p* < 0.0001).

Likewise, Cox regression indicated a highly significant constant temperature effect (Wald *χ*^2^ = 86.81, *p* < 0.0001) on germination (Table 2). Here, the likelihood of germination decreased by about 16% for every 1 °C increase in temperature above 15 °C. Linear contrasts displayed considerable differences in the likelihood of germination for all temperature combinations. However, the strongest differences resulted from comparisons against 25 °C (Table 3).

**Figure 2:**
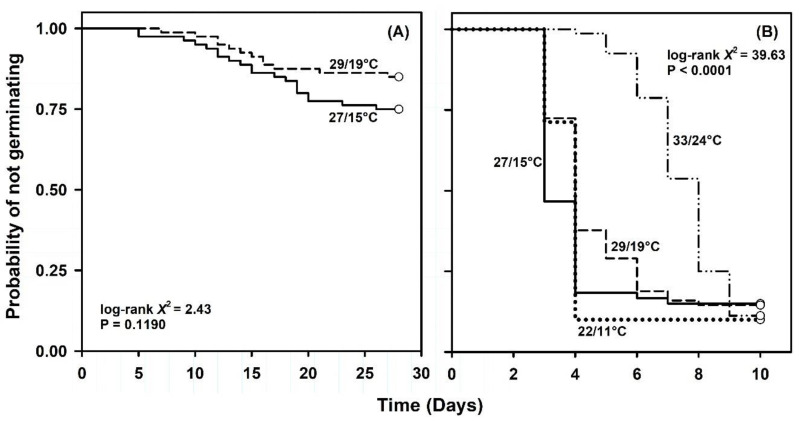
Kaplan–Meier estimates of survivor functions for *Linum arenicola* seeds exposed to (**A**) alternating temperatures that simulate the average maximum and minimum temperatures experienced throughout Florida during the winter (22/11 °C), early spring or late fall (27/15 °C), summer (33/24 °C), and early fall or late spring (29/19 °C) and (**B**) constant 22 °C after prior exposure to simulated seasonal temperatures. Pointwise 95% confidence intervals omitted for clarity.

Very few seeds germinated following exposure to simulated seasonal temperatures despite pre-sowing storage for four weeks. For example, no germination occurred at simulated summer (33/24 °C) or winter (22/11 °C) temperatures and germination did not exceed 20% following incubation at 29/19 (early fall or late spring) or 27/15 °C (late fall or early spring) conditions (Table 1). Temporal patterns indicated a considerable lack of uniformity at the temperatures where germination occurred (Figure 2A). Neither the log-rank (log-rank $\chi_{1}^{2}$ = 2.43, *p* = 0.1190) nor Cox regression detected a significant alternating temperature effect on germination (Table 2).

However, we transferred the remaining, non-germinated seeds after day 28 from simulated seasonal temperatures to constant 22 °C and a 12 h photoperiod for an additional 10 days. Interestingly, final germination after the 10-day test ranged from 85 to 90% across seeds previously exposed to simulated seasonal temperatures. Here, the germination rate peaked for seeds exposed to 27/15 °C and was about 1.3 to 2.8 times faster than at remaining temperatures (Table 1). Temporal patterns revealed a lag of 3 days for seeds previously exposed to 22/11, 27/15 or 29/19 °C. Seeds with prior exposure to simulated summer temperatures (33/24 °C) commenced germination on day 5. Germination uniformity was similar for seeds previously exposed to winter, summer, and late fall or early spring temperatures, but much less for seeds exposed to early fall or late spring temperatures (Table 1, Figure 2B). The null hypothesis that survivor functions were the same across seeds previously exposed to simulated seasonal temperatures was rejected (log-rank $\chi_{3}^{2}$ = 39.63, *p* < 0.0001).

Cox regression detected a significant alternating temperature effect on the likelihood of germination following transfer to 22 °C (Table 2). Here, the likelihood of germination decreased by about 42% for every 1° increase in simulated seasonal temperature. Linear contrasts revealed highly significant differences in the likelihood of germination for seeds first exposed to simulated winter, spring, or fall temperatures compared with summer conditions (Table 3).

### 2.2. Germination Ability in a Diurnal Photoperiod or Darkness

**Figure 3:**
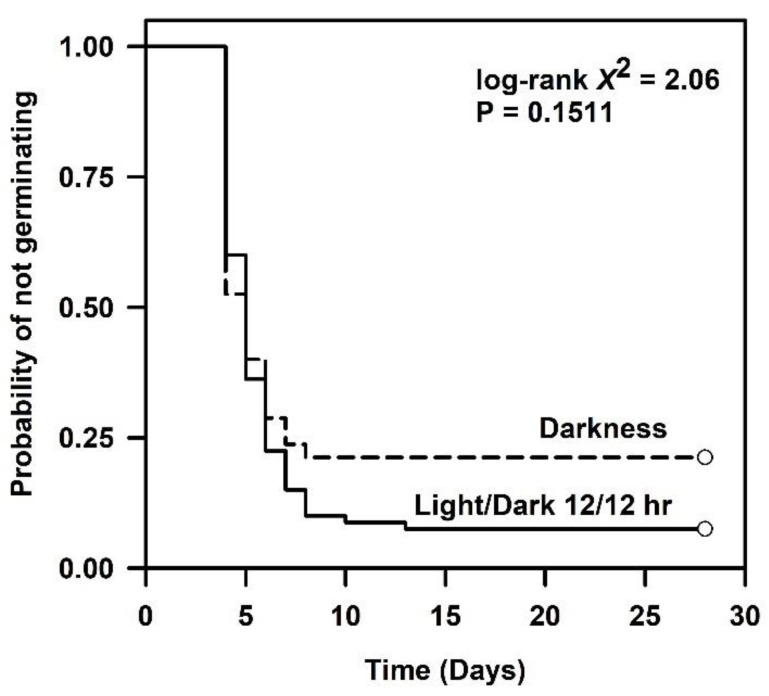
Kaplan–Meier estimates of survivor functions for *Linum arenicola* seeds exposed to 12 h alternating photoperiod or dark conditions under constant 22 °C. Pointwise 95% confidence intervals omitted for clarity.

Germination under alternating photoperiod or dark conditions at 22 °C resulted in high levels of germination (>75%). However, the final germination percentage was about 15% higher in alternating light/dark conditions compared with complete darkness (Table 1). Temporal patterns of germination remained similar for the first six days of the germination test. For example, seeds displayed the same amount of germination lag and rate under both conditions. Moreover, uniformity was similar (Figure 3, Table 1). We accepted the null hypothesis ($\chi_{1}^{2}$ = 2.06, *p* = 0.1511) that temporal patterns of germination were similar across light or dark treatments. The likelihood of germination for seeds exposed to darkness was about 81% that of seeds in an alternating photoperiod, but the effect was not significant (Table 2).

### 2.3. Germination Following a Salinity Concentration Challenge

**Figure 4:**
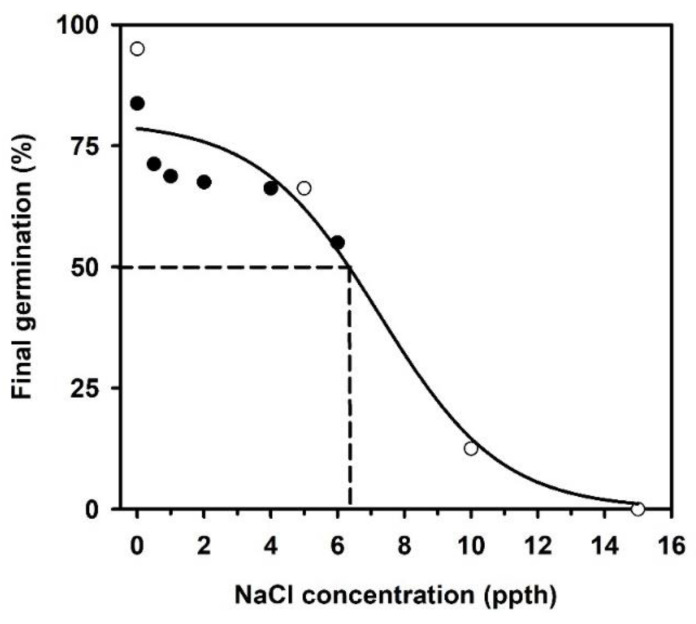
Relationships between *Linum arenicola* germination and NaCl concentration. Dotted line represents median effective concentration to reduce germination (EC_50_) and was calculated as EC_50_ = 6.34 ppth. Open circles represent the first experiment, which consisted of NaCl concentrations from 0 to 45 ppth. No germination occurred in seeds exposed to >10 ppth NaCl. Black circles represent the second experiment with salinity concentrations between 0 and 6 ppth.

Salinity concentrations affected the final percentage and rate of *Linum arenicola* seed germination. For example, final germination ranged from about 90 and 65% for seeds exposed to salinity concentrations between 0 and 5 ppth. Final germination decreased rapidly for concentrations above 5 ppth. Analysis of final germination versus NaCl concentration yielded 6.34 ppth as the median effective concentration to reduce germination (EC_50_, Figure 4).

**Figure 5:**
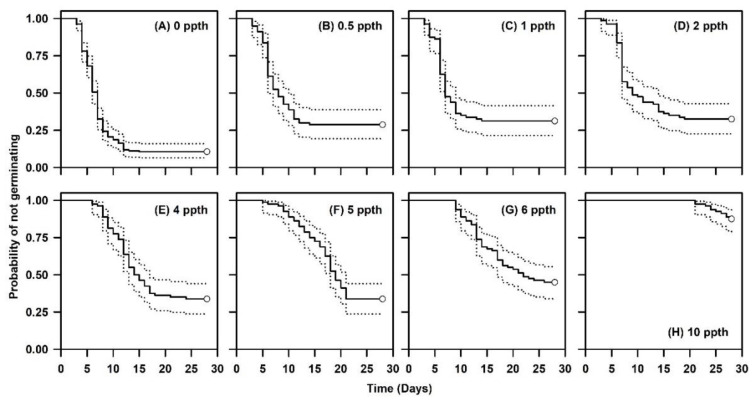
Kaplan–Meier estimates of survivor functions for *Linum arenicola* seeds exposed to various salinity levels. Dotted lines represent pointwise 95% confidence intervals. Open circles represent censored observations.

The germination rate slowed with increasing salinity concentrations and a pronounced decline occurred when the salinity concentration reached ≥4 ppth. For instance, germination rates for seed exposed to salinity levels ≥4 ppth were 1.5 to 3.5 times slower than at reduced NaCl concentrations (Table 1). Furthermore, seeds exposed to 0, 0.5, 1, or 2 ppth NaCl displayed a germination lag of about 3 days. However, the germination lag for seeds exposed to greater NaCl concentrations ranged from 5 to 21 days (Figure 5).

Temporal patterns of germination appeared similar for concentrations between 0 and 2 ppth. However, patterns started shifting upwards for concentrations ≥4 ppth, denoting that the probability of not germinating increased with the increases in salinity (Figure 5). The null hypothesis that survivor functions were the same across salinity concentrations was rejected (log-rank $\chi_{3}^{2}$ = 246.98, *p* < 0.0001).

Cox regression detected a significant effect (Wald *χ*^2^ = 174.28, *p* < 0.0001) of salinity on germination. The likelihood of germination decreased by about 22% for each 1 ppth increase in NaCl concentration (Table 2). Linear contrasts revealed that germination at 0 or 10 ppth was significantly different than germination at other salinity levels. A closer inspection of linear contrasts for germination behavior at the remaining concentrations indicates a threshold in germination response between 2 and 4 ppth. For example, significant differences in germination response are evident when the hazard ratio is greater than 1.3 (Table 4).

**Figure 6:**
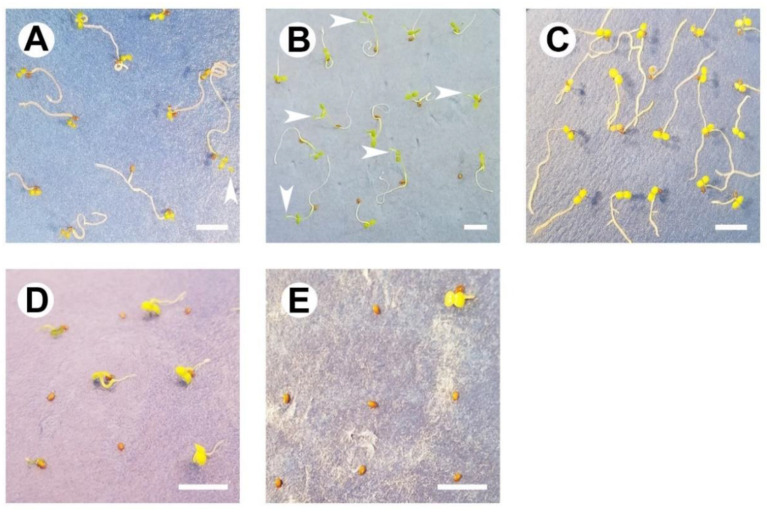
*Linum arenicola* seedlings after a 28-day germination test. Seeds were exposed to (**A**) 0, (**B**) 1, (**C**) 4, (**D**) 6, or (**E**) 10 parts per thousand NaCl. Scale bars = 5 mm. Image magnification equals (**A**) 5×, (**B**) 2×, (**C**) 5×, (**D**) 10×, and (**E**) 10×. Arrowheads denote shoots with leaves.

The resulting seedlings also displayed qualitative differences as salinity levels increased. Seedlings that developed from seeds exposed to salinity concentrations ≤4 ppth NaCl displayed intact cotyledons and elongated radicals. However, seedlings from seeds exposed to the lowest concentrations produced elongated shoots with leaves, whereas shoots were not evident for seedlings exposed to 4 ppth NaCl. Additionally, seedlings resulting from seeds exposed to 4 ppth NaCl appeared more yellow than green (Figure 6A–C). The few seedlings resulting from seeds exposed to salinity concentrations >4 ppth appeared stunted and maintained a yellow coloration in cotyledons that emerged (Figure 6D,E).

### 2.4. Germination Niche Breadths

Overall, germination niche breadth was narrow (e.g., <0.435) for seeds exposed to constant temperatures following four weeks of storage. However, when using a scaled B_*n*_ value, germination niche breadth for seeds harvested in 2018 decreased by about 19% compared with 2017 seeds, despite incubation at similar constant temperatures. Likewise, seeds receiving four weeks of storage followed by incubation at simulated seasonal temperatures displayed a narrow response breadth (e.g., <0.475). Alternatively, germination niche breadth increased by nearly 73% when seeds were transferred to constant 22 °C. Germination niche breadth was the greatest, with a value approaching 1.00, in response to the photoperiod gradient. Alternatively, germination niche breadth for seeds exposed to a salinity gradient was narrow and comparable to thermal niche breadths (Table 5).

## 3. Discussion

We examined seed functional traits and germination niche breadth of *Linum arenicola* seeds exposed to a range of thermal, photoperiodic, and salinity gradients to gain perspectives on the seed biology of this endangered species that may inform conservation decision making and assist recovery plan development. We found that fresh seeds possess non-deep physiological dormancy and the germination response of non-dormant seeds is promoted by constant rather than alternating temperatures. Non-dormant seeds do not possess an absolute light requirement for germination, but are sensitive to low levels of salinity. The narrow thermal and salinity germination niche breadths reported here suggest a specialized reproductive strategy that may require careful consideration when planning ex and in situ conservation activities.

### 3.1. Seed Functional Traits

Dormancy is a seed trait that synchronizes germination with periods when environmental conditions support a high likelihood of seedling establishment rather than simply a high likelihood of germination [24]. Seeds of *L. arenicola* are dormant at shedding and a relatively short duration experimental dormancy breaking treatment promotes germination. This suggests that seeds of *L. arenicola* most likely possess non-deep physiological dormancy, which is consistent with the class of dormancy found in the genus *Linum* [24,43]. The storage conditions (4 weeks, 22–25 °C, 30–55% relative humidity (RH)) applied to *L. arenicola* seeds in this study are adequate for inducing a dry after-ripening effect capable of alleviating non-deep physiological dormancy. Such cost-effective treatments are suggested to prepare seeds for sowing in vegetation restoration activities [44,45].

However, seeds of *L. arenicola* remaining in the field probably do not undergo dry, after-ripening on or away from the mother plant because most seeds are shed during the rainy season (i.e., May–October) and ambient relative humidity rarely falls below 50% for extended periods [36,46,47]. Instead, we suspect that *L. arenicola* seeds in the field undergo periods of warm, moist stratification that induce natural dormancy alleviation because seed shedding coincides with the wettest and warmest times of the year. For example, the ranges of monthly rainfall, average maximum temperature, and average minimum temperature for June through August (i.e., seed shedding period) in the region where *L. arenicola* occurs are about 160–225 mm, 30–35°C, and 22–23°C, respectively [48,49]. This may be one reason that seeds of *L. arenicola* require a relatively brief period (i.e., one month) of dry after-ripening. For example, Baskin and Baskin [44] assert that after-ripening at room temperature can occur in one to three months for species with seeds that naturally experience dormancy break when habitat temperatures are ≥15 °C.

Additionally, evolving a dormancy trait that requires alleviation during the wettest and warmest parts of the year makes ecological sense. First, although seeds of *L. arenicola* are shed during the wet season, rainfall can be highly variable. Moreover, intermittent (3–5 year recurrence interval) or prolonged (10–20 year recurrence interval) droughts do occur during the seed shedding period. Second, maximum temperatures regularly exceed 30–35 °C and evapotranspiration in the region is typically high during this period. Third, the ooilitic limestone rocklands on which *L. arenicola* occurs drain rapidly and contain little to no soil profile [36,37,49]. The collective effects of these abiotic factors plus the general lack of a soil layer that buffers temperature and moisture extremes can be lethal to young seedlings. For instance, the permanent wilting percentage for most non-xerophytic species corresponds to soil water potential of about −1.5 MPa [50]. Therefore, shifting germination to periods with reduced temperatures and evapotranspiration may facilitate subsequent seedling establishment. It is important to note, however, that Abiy et al. [49] detected an overall increase in annual wetness, but a decrease and an increase in wet and dry season durations, respectively.

The considerable difference in the germination capacity of *L. arenicola* seeds first exposed to a dormancy alleviation treatment then sown in constant or alternating simulated seasonal temperatures is intriguing. In this case, *L. arenicola* seeds seem to display preferential germination at constant temperatures. This contrasts with the general observation, which includes seeds of other Florida native wildflowers, of more complete and rapid germination for seeds exposed to alternating compared with constant temperatures [24,30,51,52]. Regardless, the functional significance of an optimal constant temperature may be to serve as a cue signaling an acceptable season for germination and seedling establishment [15]. The optimal constant temperature for *L. arenicola* germination, accounting for yearly differences, falls between 20 and 22 °C, which is low compared with reports for other seeds of tropical and sub-tropical species [53]. Nonetheless, this optimal temperature range is common during the late fall, winter, and early spring in southeastern Florida. Although rainfall decreases during these seasons, evapotranspiration decreases substantially as well [49]. Likewise, biotic (e.g., shrubs, forbs, grasses) and abiotic (e.g., rocks, crevices) nurse objects may facilitate germination and seedling establishment by diminishing environmental stressors such as high temperatures, low soil moisture, and high evapotranspiration. For example, nurse plants and rocks may decrease maximum temperatures by 7–11 °C [54,55,56]. Such decreases in temperatures brought about by nurse objects could represent a complete or near complete elimination of the amplitude between mean maximum and minimum alternating temperatures in the *L. arenicola* habitat. This type of facilitation, if it exists, may be important as non-dormant seeds of *L. arenicola* avoid germination and are thermo-inhibited at temperatures >25 °C and simulated summer temperatures (33/24 °C), respectively.

Alternatively, Baskin and Baskin [24] point out that mature, viable seeds exhibiting low germination (≤20%) following testing over a range of taxon-specific temperatures are most likely dormant. Further, seeds possessing non-deep physiological dormancy are either unable to germinate at any test temperature or only germinate over a very narrow temperature gradient. Seeds expressing the latter condition may be in a state of conditional dormancy. Conditional dormancy represents a series of transitional states between dormancy and non-dormancy, wherein, as dormancy loss progresses, seeds are capable of germinating over a wider range of temperatures [24]. Seeds of *L. arenicola* may be in a state of conditional dormancy at shedding, despite the application of a dormancy alleviating treatment, as evidenced by the nearly complete lack of germination at alternating simulated seasonal or warm constant temperatures, but higher levels of germination at cooler constant temperatures. Similarly, conditional dormancy was reported in seeds of *Linum catharticum* [24]. Adjustments to the current storage treatments or application of other known dormancy-breaking treatments may be necessary to thoroughly alleviate dormancy in most *L. arenicola* non-deep physiological dormancy phenotypes [24,27,57,58] and permit more complete germination at permissive temperatures. It is also possible that the after-ripening treatment imposed on *L. arenicola* seeds in this study may have functioned in such a way as to alleviate dormancy and permit germination at cooler temperatures, which represent safe thermal conditions for germination of other Florida wildflowers [51,52]. However, non-dormant, imbibed seeds exposed to warmer temperatures were not capable of germination because of thermo-inhibition. This type of post-after-ripening thermo-inhibition has been reported in other species and acts as a mechanism for non-dormant seeds to avoid germination during periods of elevated temperatures [57,59].

Light represents another important environmental factor influencing dormancy and germination. Furthermore, a light requirement for germination represents a seed trait that may function as a temporal and spatial detection mechanism that enhances seedling establishment at the proper depth in the soil profile, in relation to the nearest competitors, and after disturbance [15,24,30]. Dormancy of fresh *L. arenicola* seeds was unaffected by a constant temperature × light interaction and seeds failed to germinate regardless of exposure to light. However, the light requirement for germination may be partially or completely alleviated in seeds exposed to dry after-ripening at room temperature [24]. The role for light in dormancy break of freshly shed *L. arenicola* seeds requires more study. In contrast, non-dormant *L. arenicola* seeds were not photoblastic. These seeds germinated to high percentages (>75%) and displayed similar rates, uniformity, and temporal patterns of germination. Baskin and Baskin [24] note that the seeds of many species germinate to high proportions in light and darkness. An ability to germinate in darkness and lack of an absolute light requirement for non-dormant *L. arenicola* seeds combined with the ability to sense appropriate germination temperatures may increase the probability of seedling establishment in multiple types of safe sites.

Alterations to natural hydrological cycles, saltwater intrusion induced by sea-level rise, and inundation resulting from tropical cyclone produced storm surge often lead to sustained increases in soil salinity. Even low levels of soil salinity (e.g., ≤1 ppth) are detrimental to the germination of nearly all non-halophytic species. Moreover, the seeds of many halophytic species experience reduced germination capacity (≤10%) at salinity levels ranging from 2.8 to 3.5 ppth. Diminished germination capacity in response to salinity stress may be the result of changes in osmotic potential that decrease imbibition, interference with enzyme activity, reduced energy production, or the accumulation of toxic ions [24,30,60].

However, the evolution of seed traits that enable tolerance of varying salt concentrations functions to enable germination and seedling establishment in areas where soil salinity concentrations may be spatially and temporally elevated such as coastal or near-coastal habitats [15,24,30]. For example, germination capacity in seeds of some halophytes increases at increasing salinity concentrations as dormancy is alleviated. Yet, in other halophytes, maximum temperatures and salinity concentrations at which germination occurs also increase following dormancy break [24]. We found no such responses in non-dormant *L. arenicola* seeds exposed to increasing salinity levels and imbibed at an optimum germination temperature. In fact, the lack of germination of *L. arenicola* seeds at low salinity levels is consistent with the germination response of species not attuned to saline environments [24,30]. Furthermore, seedlings resulting from our salinity challenge appeared abnormal. These types of responses may pose challenges for *L. arenicola* seedling establishment in areas or times with slightly elevated soil salinity.

Sea level in the Florida Keys increased by approximately 2.33 (+/− 0.15) millimeters per year in the past century. Projected increases in sea level rise may drive increases in groundwater and soil salinity levels throughout southeastern Florida and the Keys [61]. Moreover, the seed-to-seedling transition represents a critical life-history change for spermatophytes and one that is highly sensitive to abiotic stress. *Linum arenicola* grows in low lying areas vulnerable to saltwater inundation or saltwater intrusion. The results presented here demonstrate that *L. arenicola* seed germination is susceptible to relatively low levels of salinity. Similarly, Wendelberger [62] found low concentration salinity-induced inhibition of germination for a suite of coastal species occurring in south Florida. Furthermore, Moghaddam et al. [63] reported that low levels of salinity (5.9 to 11.7 ppth NaCl) considerably reduced germination and subsequent seedling mass of *Linum usitatissimum*. Additionally, seedlings of *L. usitatissimum* exposed to increasing salinity appeared abnormal. So, while adult plants may survive slightly higher levels of soil salinity, reductions in the number of seed-to-seedling transitions may adversely influence population dynamics. Alterations to seedling recruitment dynamics resulting from disrupted germination patterns can be maladaptive [64].

### 3.2. Multi-Dimensional Germination Niche Breadth

The narrow and annually variable thermal germination response breadths calculated for *L. arenicola* suggest that non-dormant seeds exploit a limited range of available region-specific temperatures. Yearly variation in thermal niche breadths can be influenced by environmental conditions experienced by maternal plants. However, shifts towards broader thermal response breadths seem unlikely for *L. arenicola* given its very narrow distribution [19,23]. This is problematic as the average annual temperature in the region where *L. arenicola* grows is about 23.8 °C. This value is already above the optimum thermal range (i.e., 20–22 °C) for germination of non-dormant seeds and germination response appears sensitive to temperatures above this range. Furthermore, temperatures in south Florida have risen by 1.4 to 1.6 °C in the last century [65] and mean annual temperatures are expected to rise 3 °C or more in the next century [66]. Therefore, sustained increases to ambient temperatures have the potential to disrupt germination capacity of *L. arenicola* given the narrow thermal germination niche of this species.

Likewise, *L. arenicola* seeds display a narrow germination niche breadth in response to salinity. Although a few populations of *L. arenicola* occurring in the Florida Keys grow near the coastline of individual islands, most remaining populations do not occur along the coast. Potential salinity induced differences in germination niche breadths among island and mainland populations deserve more attention. Nonetheless, mainland populations of *L. arenicola* likely cannot exploit diverse levels of salinity for germination. The apparent reduction in *L. arenicola* seedling quality following exposure to increasing levels of salinity also highlights the need to extend niche breadth analyses beyond the germination stage. For example, Rao and Singh [21] found some variation in response breadths for several oak seedling growth parameters and linked these to regeneration ability. More attention to this type of research can elucidate the impacts of projected sea level rise on habitats near coastlines.

In contrast, non-dormant *L. arenicola* seeds displayed a very broad germination niche breadth in response to an alternating photoperiod or dark conditions when imbibed at an optimal germination temperature. Such a broad niche in terms of light availability makes ecological sense when considering the rockland habitat. First, this type of habitat consists of many exposed areas with little to no vegetative canopy or soil profile. Next, the limestone substrate is typically very rough, marked regularly with crevices and solution holes. Light may be excluded or considerably reduced in these geological features depending on the orientation and depth of crevices or solution holes in the limestone substrate. Adult *L. arenicola* plants are often found growing on open sites and emerging from solution holes [35]. Therefore, the ability to germinate in light or dark to low light conditions can be advantageous to support population expansion in considerably different microhabitats.

Overall, the germination niche breadths presented here imply a specialized regeneration strategy for *L. arenicola*. This aligns with the hypotheses of increased specialization for narrowly distributed, non-pioneer species growing in temporally stable environments such as warm, tropical, or sub-tropical regions. We envision that physical germination niches for *L. arenicola* germination must primarily provide low levels of salinity along with reductions in overall temperatures and temperature amplitude. If these conditions are satisfied, then non-dormant seeds can utilize multiple light conditions.

## 4. Materials and Methods 

### 4.1. Fruit Collection, Seed Handling, and General Methods

We collected (FDACS-DPI Collection Permit # 1308) dry capsules of *Linum arenicola* in June 2017 and 2018 during the period of natural dehiscence from over 25 plants growing in 14 sites located along a 200–300 m segment of the L-31E levee (Miami-Dade Co., FL, USA, Figure S1). We consider plants distributed along the L-31E canal as one population and refer to these collections as the 2017 and 2018 lots, respectively. We either (1) removed seeds from 2017 fruits within 2 days of collection or (2) stored fruits from 2017 and 2018 in paper envelopes within the lab (22 to 25 °C and 30 to 55% RH) for 4 weeks prior to seed removal and experimentation. We rinsed seeds with distilled water for 1–2 min following extraction then immediately sowed seeds. We used radicle protrusion ≥1 mm as our marker of germination for all experiments described below and conducted germination counts daily for 28 days.

Seed availability represents a persistent challenge for any aspect of seed biology research related to threatened plant species. Our seed supply was limited for both collection years. Therefore, we calculated adequate sample sizes for the experiments described below utilizing methods outlined in Schoenfeld and Richter [67] and Peduzzi et al. [68]. Furthermore, the limited number of seeds precluded optimization of post-germination viability assays (e.g., tetrazolium staining, seed dissection, and embryo analysis). Non-germinated seeds remaining at the end of experiments appeared intact. Therefore, we calculated germination percentages on the total number of seeds for a given experiment.

### 4.2. Assessing Germination Capacity under Constant or Simulated Seasonal Temperatures

We randomly selected 320 seeds from the 2017 lot and divided this working sample into eight samples of 40 seeds each. We then randomly assigned each sample to one of eight constant temperature treatments between 5 and 40 °C achieved on a thermo-gradient table (Model 5010.00, Seed Processing Holland BV, Enkhuizen, The Netherlands). We divided each sample into four sub-samples of 10 seeds each and sowed these on blotter paper (Blue Steel, Anchor Paper, St. Paul, MN, USA) moistened with 15 mL of distilled, deionized water within 15 × 90 mm Petri dishes. We repeated constant temperature experiments with the 2018 seed lot. However, we exposed seeds to temperatures between 10 and 35 °C using 5 °C increments. In this experiment, we used four sub-samples of 20 seeds and tested germination within programmable chambers (I-30VL, Percival Scientific, Inc., Perry, IA, USA) each set to one temperature. We exposed all seeds here and in subsequent experiments to a 12 h daily photoperiod provided by cool white fluorescent lamps. Photosynthetic photon flux density at seed level reached 52 ± 5 and 57 ± 7 umol m^−2^ s^−1^ on the thermo-gradient table and within the chambers, respectively.

We used the 2018 seed lot to examine seed response to simulated seasonal temperatures. We randomly selected 320 seeds and divided this into four samples of 80 seeds each. We then randomly assigned each sample to one of four simulated seasonal diel (12/12 h) temperatures: 22/11, 27/15, 29/19, or 33/24 °C. We derived simulated seasonal temperatures from average monthly maxima and minima collected by the Southeast Regional Climate Center over a 30-year period at sites across Florida. Our temperatures simulate winter (22/11 °C), late fall or early spring (27/15 °C), early fall or late spring (29/19 °C), or summer (33/24 °C). We divided each sample into four sub-samples of 20 seeds and sowed these as described above. Illumination coincided with higher temperatures in the alternating regime. Germination was limited for seeds exposed to simulated seasonal temperatures. Therefore, we transferred any remaining non-germinated seeds to a chamber set at 22 °C and with a 12 h photoperiod.

### 4.3. Evaluating Germination Ability with a Diurnal Photoperiod or Darkness

We compared the ability of *L. arenicola* seeds to germinate following exposure to a diurnal photoperiod (12 h light/12 h dark) or darkness with seeds from the 2018 seed lot. We randomly selected 160 seeds and divided this into two samples of 80 seeds each. We then randomly assigned samples to treatments and sowed four sub-samples of 20 seeds each on blotter paper as described previously. We wrapped dishes for the dark treatments with two layers of aluminum foil immediately following seed sowing and exposed all seeds to constant 22 °C for the duration of the experiment. We checked for germination in the dark treatments only when we observed germination occurring in the treatments exposed to light. We examined dishes at night with indirect light provided by fluorescent lamps about 2 m from the seeds and exposed seeds to incidental light for about 30 s during observations.

### 4.4. Measuring Germination Following a Salinity Challenge

We randomly selected 800 seeds from the 2018 lot and then divided this working sample into 10 samples of 80 seeds each. We randomly assigned each sample to one of 10 NaCl concentrations: 0, 5, 10, 15, 20, 25, 30, 35, 40, and 45 ppth made with distilled, deionized water. We divided each sample into four sub-samples of 20 seeds each, sowed these on blotter paper moistened with 15 mL of the appropriate NaCl solution within 15 × 90 mm Petri dishes and incubated seeds at constant 22 °C and a 12 h daily photoperiod achieved on the thermo-gradient table. We observed that germination decreased considerably between the control and 5 ppth, while no germination occurred at concentrations above 10 ppth. Therefore, we repeated the experiment using NaCl concentrations of 0, 0.5, 1, 2, 3, 4, and 6 ppth to clarify the germination response at lower salinity levels.

### 4.5. Estimating Germination Niche Breadths

We computed germination niche breadth (B_*n*_) for temperature, light, and salinity gradients by using a normalized version of the reciprocal of the Simpson index [20,69].

(1) $$B_{n}=\frac{1}{\left(R\underset{i}{\sum p_{i}^{2}}\right)}$$

The variable *p_i_* equals the proportion of seed germination in state *i* (i.e., treatment) and *R* is the total number of states. *R* values for fresh (0 weeks of storage) and after-ripened seeds harvested in 2017 equaled eight (6, 11, 17, 22, 25, 31, 36, and 40 °C) and nine (4 weeks storage at about 23 °C followed by incubation at 6, 11, 17, 22, 25, 31, 36, and 40 °C), respectively. *R* values for seeds harvested in 2018 and then exposed to constant temperatures, simulated seasonal temperatures, photoperiods, and salinity equaled seven (4 weeks storage at about 23 °C followed by incubation at 10, 15, 20, 25, 30, and 35 °C), four (22/11, 27/15, 29/19, and 33/24 °C), five (22/11, 27/15, 29/19, and 33/24 °C followed by transfer to 22 °), two (12/12 h photoperiod and darkness), and fifteen (0, 0.5, 1, 2, 4, 5, 6, 10, 15, 20, 25, 30, 35, 40, and 45 ppth NaCl). B_*n*_ scales from 1/*R* to 1.0 with values close to 1/*R* and 1.0 denoting a narrow and wide niche, respectively [69].

We compared B_*n*_ values for (1) after-ripened seeds harvested in 2017 and 2018 and (2) seeds harvested in 2018 and then exposed to simulated seasonal temperatures or simulated seasonal temperatures followed by incubation at 22 °C to assess temporal and thermal variation on germination niche breadth. However, values of 1/R differ among the experiments. This leads to differences in scales of measurement. Therefore, we normalized B_*n*_ values for after-ripened seeds harvested in 2018 to the scale for after-ripened seeds harvested in 2017. Likewise, we normalized B_*n*_ values for seeds transferred to 22 °C following exposure to simulated seasonal temperatures to the scale for seeds exposed only to simulated seasonal temperatures. We normalized B_*n*_ values using a feature scaling formula:

(2) $$X_{norm}=\left(b-a\right)\cdot \left[\left(x-y\right)/\left(z-y\right)\right]+a$$

where *a* is the minimum value of the range to normalize to, *b* is the maximum value of the range to be normalized to, *x* is the value to be normalized, *y* is the minimum value of the range to be normalized, and *z* is the maximum value of the range to be normalized.

### 4.6. Data Analyses

Several authors describe the benefits and appropriateness of using time-to-event analyses for evaluation of germination data [52,70,71]. We utilized non- and semi-parametric time-to-event analyses to evaluate temporal patterns of germination and construct Cox regression models. Germination was the event of interest. We used the LIFETEST procedure to generate Kaplan–Meier estimates of survivor functions and then test the null hypothesis that survivor functions were the same across treatments by calculating the log-rank statistic. We generated quartile estimates of germination from the product-limit survival estimates. Quartile estimates correspond to the smallest event times such that the probability of germinating earlier is greater than 0.25, 0.50, or 0.75. We calculated the germination rate as the inverse of median germination time (1 × *t*_50_^−1^). We used the exact method to account for large proportions of tied event times and included a time-dependent covariate as necessary to accommodate non-proportionality when constructing Cox regression models within the PHREG procedure. We used to interpret hazard ratios (i.e., likelihood of germination) for quantitative covariates (e.g., temperature, salinity). We interpreted the hazard ratio for qualitative covariates (i.e., alternative photoperiod vs. darkness) as the ratio of hazard for seeds exposed to darkness to those receiving alternating photoperiod [72]. We conducted semi- and non-parametric analyses in SAS (v 9.4, SAS Institute Inc., Cary, NC, USA).

(3) 100 (Hazard Ratio−1)

We evaluated the relationship between final germination and salinity concentration for our combined data set using non-linear regression. We fit a three-parameter sigmoid relationship using the Global Fit method with all parameters shared in SigmaPlot (v. 12.0, Systat Software Inc., San Jose, CA, USA).

## 5. Conclusions and Implications for Conservation

Mature seeds of *L. arenicola* express non-deep physiological dormancy upon shedding and a short period of after-ripening is necessary to promote germination. Seed storage in an air-conditioned room for about 4 weeks meets this requirement. Germination in the field or greenhouse will most likely occur in light or darkness after a period of dormancy alleviation and when average substrate temperatures are near 22 °C. However, other abiotic factors such as soil salinity must be considered when contemplating the potential for germination. We suspect that germination and subsequent seedling establishment would be reduced following exposure to salt concentrations >2 ppth and temperatures that deviate from the optimum. Managers can expect relatively low levels of germination for *Linum arenicola* seeds when soil salinity exceeds 4 ppth. Furthermore, seeds in the field may need to encounter spatial features in rockland habitats that limit high temperatures and temperature fluctuations.

The specialized regeneration strategy for *L. arenicola* suggests that management activities such as assisted migration may be challenging, and efforts may need to focus other actions such as inter-situ conservation for this endemic species. We suggest further research on the following: (1) the implications of secondary dispersal to and facilitation of germination by nurse objects; (2) the influence of spectral composition and light intensity on germination; (3) light × temperature interactions on dormancy alleviation and germination; and (4) temperature × salinity interactions on germination. Further analysis on germination and seedling establishment niche breadth using multiple niche dimensions is also warranted. These lines of investigation can paint a more informative picture on the germination ecology of threatened species. This is crucial because a driving goal of many species-level conservation programs is to eventually provide conditions for unaided population stability and/or growth.

## Figures and Tables

**Table 1:** The total number (*n*) of *Linum arenicola* seeds exposed to various experimental treatments and pertinent germination descriptors. Alternating temperatures simulate the average daily maxima and minima for the winter (22/11 °C), early spring or late autumn (27/15 °C), early autumn or late spring (29/19 °C), or summer (33/24 °C) throughout Florida. Salinity measurements are in parts per thousand (ppth) NaCl. Kaplan–Meier survival estimates were used to determine the time in days to reach 25, 50, or 75% germination. Germination rate was calculated as 1/*t*_50_. Germination uniformity (U) was calculated as U = *t*_75_ − *t*_25_. CI, confidence interval.

Treatment	*n*	Final Germination (%)	Percentile Estimates (95% CI)			Germination Rate (1/*t*_50_)	U
			*t*_25_	*t*_50_	*t*_75_		
Constant temperature (°C)							
2017 (fresh)							
6	40	0.0	-	-	-	-	-
11	40	0.0	-	-	-	-	-
17	40	0.0	-	-	-	-	-
22	40	0.0	-	-	-	-	-
							
25	40	0.0	-	-	-	-	-
31	40	0.0	-	-	-	-	-
36	40	0.0	-	-	-	-	-
40	40	0.0	-	-	-	-	-
2017 (post-storage)							
6	40	0.0	-	-	-	-	-
11	40	62.5	14.0 (13, 19)	24.0 (17, -) ^x^	- ^y^	0.04	-
17	40	85.0	8.0 (6, 9)	9.5 (9, 10)	10.0 (10, -)	0.10	2.0
22	40	100.0	6.0 (5, 6)	7.0 (6, 7)	8.0 (7, 9)	0.14	2.0
25	40	90.0	5.5 (5, 7)	7.5 (6, 9)	10.0 (8, 10)	0.10	4.5
31	40	0.0	-	-	-	-	-
36	40	0.0	-	-	-	-	-
40	40	0.0	-	-	-	-	-
2018 (post-storage)							
10	80	0.0	-	-	-	-	-
15	80	97.5	6.0 (-, -)	6.0 (6, 7)	9.0 (7, 11)	0.16	3.0
20	80	98.7	5.0 (-, -)	5.0 (-, -)	7.0 (6, 8)	0.20	2.0
25	80	25.0	-	-	-	-	-
30	80	0.0	-	-	-	-	-
35	80	0.0	-	-	-	-	-
Simulated seasonal temperatures ^z^ (°C)							
Prior to transfer to 22 °C							
33/24 (summer)	80	0.0	-	-	-	-	-
29/19 (early fall or late spring)	80	15.0	-	-	-	-	-
27/15 (late fall or early spring)	80	25.0	-	-	-	-	-
22/11 (winter)	80	0.0	-	-	-	-	-
Following transfer to 22 °C							
33/24 (summer)	80	88.8	7 (6, 7)	8 (7, 8)	8 (8, 9)	0.12	1
29/19 (early fall or late spring)	68	86.8	3 (3, 4)	4 (-, -)	6 (5, 8)	0.25	3
27/15 (late fall or early spring)	60	85.0	3 (-, -)	3 (3, 4)	4 (4, -)	0.33	1
22/11 (winter)	80	90.0	3 (3, 4)	4 (-, -)	4 (-, -)	0.25	1
Photoperiod ^z^							
Darkness	80	78.60	4 (-, -)	5 (4, 6)	7 (6, -)	0.20	3
12 h Photoperiod	80	92.50	4 (-, -)	5 (4, 5)	6 (6, 7)	0.20	2
Salinity ^z^ (ppth NaCl)							
0	160	89.37	5.0 (4, 5)	7.0 (6, 7)	8.0 (8, 11)	0.14	3.0
0.5	80	71.25	6.0 (5, 6)	8.0 (6, 10)	-	0.12	-
1	80	68.75	6.0 (-, -)	7.0 (7, 9)	-	0.14	-
2	80	67.50	7.0 (6, 7)	9.0 (7, 14)	-	0.11	-
4	80	66.25	11.0 (9, 12)	14.5 (13, 17)	-	0.07	-
5	80	66.25	14.5 (12, 17)	19 (18, 21)	-	0.05	-
6	80	55.00	13.0 (12, 17)	22 (17, -)	-	0.04	-
10	80	12.50	-	-	-	-	-
15	80	0.0	-	-	-	-	-
20	80	0.0	-	-	-	-	-
25	80	0.0	-	-	-	-	-
30	80	0.0	-	-	-	-	-
35	80	0.0	-	-	-	-	-
40	80	0.0	-	-	-	-	-
45	80	0.0	-	-	-	-	-

^x^ Calculated percentile estimate and missing upper or lower confidence interval are identical, denoting lack of variation between values. Note also the vertical portions of step function in Figure 1, Figure 2, Figure 3 and Figure 4. ^y^ The Kaplan–Meier estimator for these data did not reach a failure probability >0.25, 0.50, or 0.75. Refer also to the final germination percentage column. ^z^ Experiment utilized seeds harvested in 2018.

**Table 2:** Summary of Cox models for *Linum arenicola* seeds exposed to various constant temperatures, simulated seasonal temperatures, and salinity concentrations. Alternating temperatures simulate the average daily maxima and minima for the winter (22/11 °C), early spring or late autumn (27/15 °C), early autumn or late spring (29/19 °C), or summer (33/24 °C) throughout Florida.

Experiment	Model Covariates	Coefficient β	SE of βi	Wald *χ*^2^	*p*	Hazard Ratio, e(βi)
2017 harvest (constant 11, 17, 22, 25 °C)						
	Temperature	0.412	0.068	36.688	<0.0001	1.510
	Temperature × day	−0.029	0.007	17.603	<0.0001	0.971
2018 harvest (constant 15, 20, 25 °C)						
	Temperature	−0.174	0.019	86.811	<0.001	0.840
Simulated seasonal temperatures (29/19 and 27/15 °C)						
	Temperature	−0.281	0.183	2.369	0.1238	0.755
Simulated seasonal temperatures followed by 22 °C (33/24, 29/19, 27/15, 22/11 → 22 °C)						
	Temperature	−0.546	0.060	82.460	<0.0001	0.579
	Temperature × day	0.101	0.014	54.451	<0.0001	1.106
Salinity (0, 0.5, 1, 2, 4, 5, 6, 10 ppth NaCl)						
	Salinity	−0.25	0.019	174.279	<0.0001	0.778
	Salinity × day	0.045	0.004	128.117	<0.0001	1.047

**Table 3:** Linear contrasts comparing the slope coefficients for *Linum arenicola* seed germination following exposure to constant or simulated seasonal temperatures. Alternating temperatures simulate the average daily maxima and minima for the winter (22/11 °C), early spring or late autumn (27/15 °C), early autumn or late spring (29/19 °C), or summer (33/24 °C) throughout Florida.

Comparison	df	Wald *χ*^2^	*p*
2017 (constant 11, 17, 22, 25 °C)			
11 vs. 17	1	18.591	<0.0001
11 vs. 22	1	74.133	<0.0001
11 vs. 25	1	36.859	<0.0001
17 vs. 22	1	29.921	<0.0001
17 vs. 25	1	3.687	0.0548
22 vs. 25	1	14.147	0.0002
2018 (constant 15, 20, 25 °C)			
15 vs. 20	1	21.000	<0.0001
15 vs. 25	1	91.678	<0.0001
20 vs. 25	1	145.054	<0.0001
Simulated seasonal temperatures followed by 22 °C (33/24, 29/19, 27/15, 22/11 → 22 °C)			
22/11 vs. 27/15	1	0.180	0.6712
22/11 vs. 29/19	1	4.357	0.0368
22/11 vs. 33/24	1	34.920	<0.0001
27/15 vs. 29/19	1	2.316	0.1281
27/15 vs. 33/24	1	25.780	<0.0001
29/19 vs. 33/24	1	13.514	0.0002

**Table 4:** Hazard ratios with associated *p*-values in parentheses for single degree of freedom linear contrasts of *Linum arenicola* germination following exposure to increasing salinity concentrations.

Salinity (ppth NaCl)								
	**0**	**0.5**	**1**	**2**	**4**	**5**	**6**	10
0	-	2.0 (<0.0001)	2.1 (<0.0001)	2.6 (<0.0001)	3.4 (<0.0001)	3.9 (<0.0001)	4.8 (<0.0001)	25.5 (<0.0001)
0.5		-	1.0 (0.8284)	1.3 (0.2100)	1.7 (0.0067)	1.9 (0.0007)	2.4 (<0.0001)	12.6 (<0.0001)
1			-	1.2 (0.3037)	1.6 (0.0134)	1.8 (0.0016)	2.3 (<0.0001)	12.1 (<0.0001)
2				-	1.3 (0.1486)	1.5 (0.0338)	1.9 (0.0023)	9.9 (<0.0001)
4					-	1.1 (0.4982)	1.4 (0.0944)	7.5 (<0.0001)
5						-	1.2 (0.3041)	6.6 (<0.0001)
6							-	5.3 (<0.0001)
10								-

**Table 5:** Germination niche breadth (B_*n*_) for *Linum arenicola* seeds exposed to multiple environmental factors.

Seed Collection Year	Environmental Gradient	1/*R*	B_*n*_	Scaled ^Z^ B_*n*_
2017	Constant temperatures	0.125	-	-
	After-ripening, constant temperatures	0.111	0.433	-
2018	After-ripening, constant temperatures	0.143	0.352	0.328
	Simulated seasonal temperatures	0.250	0.471	-
	Simulated seasonal temperatures, transfer to 22 °C	0.200	0.800	0.813
	Photoperiod	0.500	0.993	-
	Salinity	0.067	0.480	-

^Z^ Values normalized using a feature scaling formula: *X*_norm_ = (*b* − *a*) ∙ [(*x* − *y*)/(*z* − *y*)] + *a*, where *a* is the minimum value of the range to normalize to, *b* is the maximum value of the range to be normalized to, *x* is the value to be normalized, *y* is the minimum value of the range to be normalized, and *z* is the maximum value of the range to be normalized.

## Supplementary Materials

The following are available online at https://www.mdpi.com/2223-7747/9/11/1493/s1, Figure S1. The L-31E canal (Miami-Dade County, Florida) in relation to the (A) extreme southeastern peninsula of Florida, (B) and (C) proximity to Biscayne Bay, and (D) adjacent levee structure where *Linum arenicola* occurs. FCG = flood control gate.
