# Supplementary figures and images for: Enhancing Conservation of a Globally Imperiled Rockland Herb (Linum arenicola) through Assessments of Seed Functional Traits and Multi-Dimensional Germination Niche Breadths
Source: Plants (Basel). 2020 Nov 5;9(11):1493. doi: 10.3390/plants9111493 (PMC7694399; doi:10.3390/plants9111493)

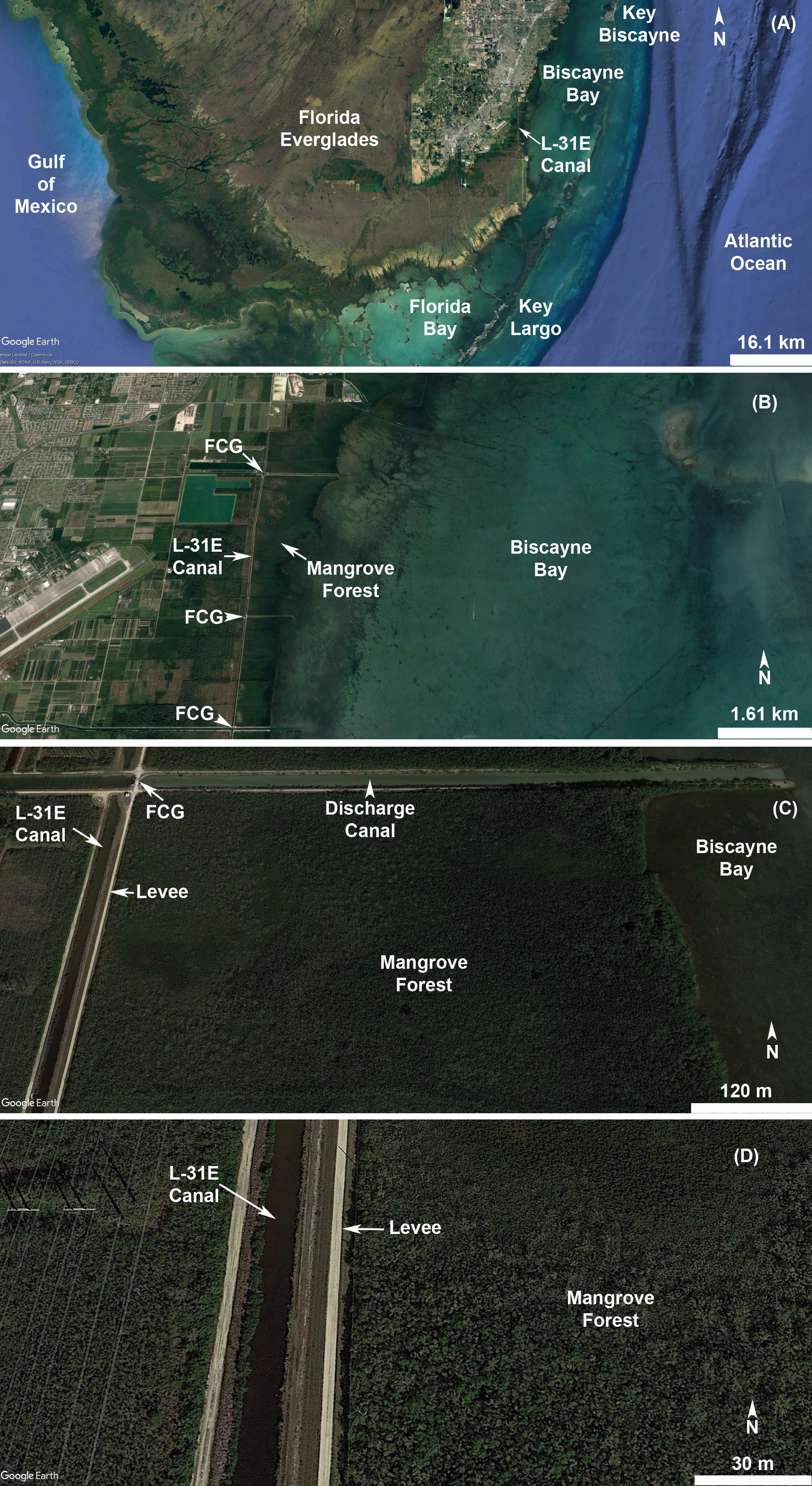

Supplement: Supplementary file 1 [file plants-09-01493-s001.zip › Figure_S1.tif]
